# Generation of a Collision Cross Section Library for Multi-Dimensional Plant Metabolomics Using UHPLC-Trapped Ion Mobility-MS/MS

**DOI:** 10.3390/metabo10010013

**Published:** 2019-12-24

**Authors:** Mark Schroeder, Sven W. Meyer, Heino M. Heyman, Aiko Barsch, Lloyd W. Sumner

**Affiliations:** 1 Department of Biochemistry, Bond Life Sciences Center, University of Missouri, Columbia, MO 65211, USA; mjsfp9@mail.missouri.edu; 2 Solutions Development, Bruker Daltonics, 28359 Bremen, Germany; Sven.Meyer@bruker.com (S.W.M.); Heino.Heyman@bruker.com (H.M.H.); Aiko.Barsch@bruker.com (A.B.)

**Keywords:** collision cross section, CCS, trapped ion mobility spectrometry, TIMS, mass spectrometry, natural products, adducts, metabolomics

## Abstract

The utility of metabolomics is well documented; however, its full scientific promise has not yet been realized due to multiple technical challenges. These grand challenges include accurate chemical identification of all observable metabolites and the limiting depth-of-coverage of current metabolomics methods. Here, we report a combinatorial solution to aid in both grand challenges using UHPLC-trapped ion mobility spectrometry coupled to tandem mass spectrometry (UHPLC-TIMS-TOF-MS). TIMS offers additional depth-of-coverage through increased peak capacities realized with the multi-dimensional UHPLC-TIMS separations. Metabolite identification confidence is simultaneously enhanced by incorporating orthogonal collision cross section (CCS) data matching. To facilitate metabolite identifications, we created a CCS library of 146 plant natural products. This library was generated using TIMS with N_2_ drift gas to record the ^TIMS^CCS_N2_ of plant natural products with a high degree of reproducibility; i.e., average RSD = 0.10%. The robustness of ^TIMS^CCS_N2_ data matching was tested using authentic standards spiked into complex plant extracts, and the precision of CCS measurements were determined to be independent of matrix affects. The utility of the UHPLC-TIMS-TOF-MS/MS in metabolomics was then demonstrated using extracts from the model legume *Medicago truncatula* and metabolites were confidently identified based on retention time, accurate mass, molecular formula, and CCS.

## 1. Introduction

Metabolomics is a revolutionary systems biology tool for understanding plant metabolism and elucidating gene function [1,2,3]. The continual refinement, increasing scope and larger-scale have led to the evolution of modern plant metabolomics, which has matured as a valuable tool for advancing our understanding of plant biology and physiology [2,4,5,6]. Although the vast utility of metabolomics is well documented in the literature, its full scientific promise has not yet been realized due to multiple technical challenges. These challenges have been reviewed by the metabolomics community and a set of grand challenges identified by the Plant, Algae, and Microbial Metabolomics Research Coordination Network [7]. At the top of these grand challenges are accurate and confident chemical identification of all observable metabolites and increasing the metabolome depth of coverage. Here, we propose a multi-dimensional, combinatorial solution for both using UHPLC-trapped ion mobility coupled to mass spectrometry (UHPLC-TIMS-TOF-MS).

Historically, most metabolomics platforms have relied on one-dimensional separations by gas or liquid chromatography (GC or LC) coupled to mass spectrometry (MS). The separation potential of these chromatographic methods can be theoretically defined by its peak capacity (*P*), which is a common metric used to define the separation potential of a chromatographic method [8] (Equation (1)).

(1) P=1+tg(1n)∑1nw,

*P* is peak capacity, *t_g_* is the total gradient time, *n* is the number of peaks used to sum the peak widths *w* and the peak widths are calculated at the base measured at 4σ. Essentially, one can estimate *P* by dividing the total gradient time by the average peak width. 

The peak capacity for LC systems has evolved over the past 50 years to an upper limit of approximately 500–1000 for current UHPLC systems. Peak capacity increases have been associated with increasingly higher pressure pumps and smaller particles [9]. Thus, one can reasonably estimate that maximum peak capacities will continue to grow to approximately 1000–2000 over the next 50 years based upon historical linear trajectories. However, historical linear increases will never get us in a timely manner to peak capacities of 100,000 or more which are needed to adequately provide the depth-of-coverage necessary for truly comprehensive metabolomics. Such depth-of-coverage can only be potentially achieved through multi-dimensional separations. The peak capacity of multi-dimensional systems is the product of the peak capacities of the two or more dimensions and not the linear sum. Thus, many have utilized two-dimensional GC × GC [10,11] and two-dimensional LC x LC as a means to increased peak capacities for complex mixture analyses [11,12]. However, most 2D GC or LC systems are not optimally timed. Ideally one would prefer a minimum of ten 2D separations across each peak in the first dimension. Thus, a 6 s peak (measured at 4σ) in a 1D UHPLC, would ideally be analyzed in the second-dimension using separations of 600 ms or less. Most 2D GC or LC methods cannot achieve this speed and typically operate in the 10 s of seconds for the second dimensional separations [12]. However, second dimensional ion mobility separations are achievable in this time frame [13,14], and amongst the available technologies, trapped ion mobility spectrometry offers higher resolving powers and peak capacities than traditional drift tube ion mobility [15,16]. We have coupled UHPLC to trapped ion mobility spectrometry and tandem mass spectrometry (UHPLC-TIMS-TOFMS/MS) to yield a multi-dimensional metabolite profiling solution for increasing our metabolome depth of coverage. We estimate the peak capacity of this system to be 2 × 10^9^ based upon [(P_UHPLC_ ~ 500) × (P_TIMS_ ~ 20) × (P_QTOF-MS_ ~ 200,000)]. Mass spectrometry offers substantial peak capacities; however, there are large numbers of isobaric and isomeric compounds in biology that cannot be resolved by mass spectrometry. More specifically, plants contain many specialized metabolites, e.g., flavonoids and terpenoids, that are of interest for biological and industrial purposes [17,18,19]. These compound classes contain many isomers that can be difficult to distinguish based upon mass alone. Thus, the second dimensional peak capacity of TIMS offers additional opportunities for the differentiation of such isobaric and isomeric compounds.

The number one grand challenge of metabolomics is the confident identification of all metabolites. According to the Metabolomics Standards Initiative (MSI) [20], confident metabolite identifications are achieved through matching of two or more orthogonal experimental data values with that of authentic standards [21,22]. This commonly includes matching of chromatographic retention time/index and mass spectra, but unfortunately, a limited number of mass spectral and retention time libraries exist for identification [23,24]. Fortunately, identification confidence can be further enhanced by matching additional orthogonal data such as collision cross section values (CCS). 

**Figure 1:**
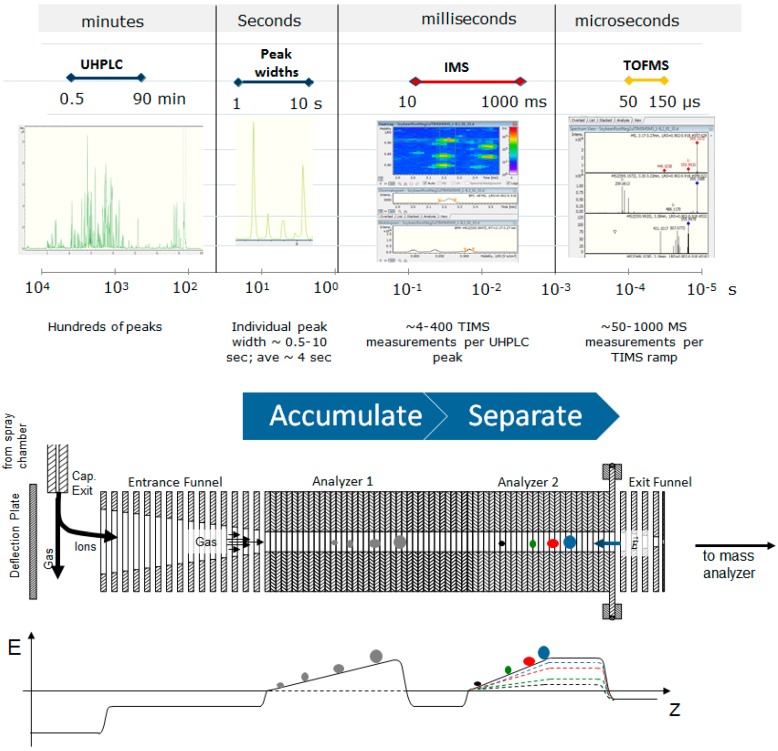
Trapped ion mobility spectrometry (TIMS) is temporally well aligned with UHPLC as a second dimensional separation technology. TIMS also offers higher resolution and a high duty cycle. Ions from the source are pushed through the ion tunnel by a drift gas and opposed by an electric field gradient, in effect trapping them where those forces are equal on the ion. The two stages of the TIMS allow for accumulation of ions in analyzer 1 while separation and mobility measurements take place in analyzer 2. This results in a near 100% duty cycle. As the electric field gradient is lowered, the ions are released from the second analyzer towards the time-of-flight (TOF) detector.

Ion mobility spectrometry (IMS) separates metabolites based upon size and charge, and it measures the mobility of an ion in a drift gas [13,25]. Commercial IMS instruments tend to use N_2_ gas, but He, CO_2_, and other gases can also be used. Ion mobility data are plotted as ion mobility spectra and are used to calculate collision cross sections (CCS), which are the average spatial size and shape of gas phase compounds [26]. Mobility is inversely related to CCS, and a small mobility represents a large CCS value. Therefore, mobility data are often presented as an inverse reduced mobility (1/K_0_) [15]. CCS is a physical property of a compound measured using a specific drift gas, temperature and pressure. Thus, the ion mobility community has recently published recommendations for reporting ion mobility metadata [27] to help ensure that the many variables that influence K_0_ and CCS measurements are reported. Trapped ion mobility spectrometry (TIMS; Figure 1) circumvents some of the previous challenges of IMS and offers higher resolutions of approximately 200 with much smaller instrumental size relative to drift tube IMS [28,29]. CCS values can also be acquired with very high reproducibility using TIMS. Thus, we have opted to use TIMS in our instrumental metabolomics approach. 

Here, we report the creation of a CCS library for improved identification of compounds in plant metabolomics by adding another dimension of chemical parameter matching. We used UHPLC-TIMS-QTOF-MS/MS in negative electrospray ionization mode to calculate CCS values for inclusion in the library. A substantial number of plant natural products include phenolic and carboxy substituents that ionize very well in the negative ESI, which also has the advantage of lower chemical noise that ultimately results in better MS S/N ratios for these compounds. Data generated were highly reproducible, and we describe additional methods to accurately annotate ions observed in the ion mobility spectrum. Reported CCS measurements were robust during coelution and also in the presence of a complex plant matrix. The utility of the CCS library for the identification of plant natural products based upon accurate mass, R_t_, and CCS was demonstrated using an extract from the model legume *Medicago truncatula* and data evaluation using Bruker MetaboScape software.

## 2. Results and Discussion

### 2.1. CCS Library

A Bruker timsTOF Pro™ and concurrent gas composed of 99.4% N_2_ and 0.6% O_2_ from a Parker Balston Model N2-80A membrane based nitrogen generation system were used to record ^TIMS^CCS_N2_ values for 146 authentic plant natural products mostly consisting of flavonoids, glycosylated flavonoids, isoflavonoids, triterpenes, and glycosylated triterpenes. The major ion species observed were [M-H]^−^ and the data for this group were as follows. The masses of authentic standards sampled ranged from 210 to 826 Da. CCS measurements ranged from 141.26 to 300.86 Å^2^. The UHPLC gradient separation was 35 min and the average and standard deviation retention times were 13.19 ± 7.79 min. Multiple adducts and aggregate ions were observed. Those that were identified included [M-H]^−^, [M-H+HCO_2_H]^−^, [M-H+Na+HCO_2_^-^]^−^, [2M-H]^−^, and [M-3H]^−^. The cumulative data and all proposed ion annotations are included in Tables S1 and S2 (Excel File) and CCS values plotted in Figure S1.

#### 2.1.1. Statistics and Reproducibility

**Figure 2:**
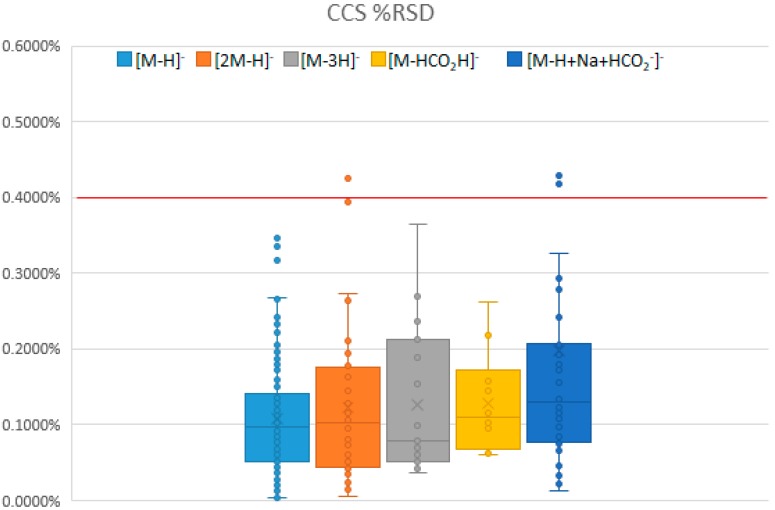
The percent relative standard deviation (RSD) for various collision cross section (CCS) measurements organized by ion annotations: [M-H]^−^ (*n* = 146), [2M-H]^−^ (*n* = 44), [M-3H]^−^ (*n* = 19), [M-H+HCO_2_H]^−^ (*n* = 10), and [M-H+Na+HCO_2_^−^]^−^ (*n* = 37). The average relative standard deviation of triplicate measurements for all measured authentic standards was 0.1026%. There were greater deviations associated with larger adducts i.e., [M-H+Na+HCO_2_^−^]^−^ and [2M-H]^−^.

The CCS values, average, standard deviation, and relative standard deviation (RSD) were recorded for triplicate analyses of each authentic standard and are summarized in Figure 2. The [M-H]^−^ was the primary ion of interest. The minimum standard devation was 0.0058 Å^2^, the average was 0.1988 Å^2^ (±0.1469 Å^2^), the maximum was 0.7401 Å^2^ for all the triplicate analyses. The minimum RSD was 0.0037%, the average RSD was 0.1075% (±0.0720%), and the maximum RSD was 0.3459%, for all triplicate analyses of [M-H]^−^ ions. Major ions are all within the strictest range of highly reproducible measurements recently published by Stow et al. [29]. The high reproducibility of the reported data illustrates the precision of the acquired CCS data and their use for compound identification. 

Calibration of the TIMS using a primary standard is a necessary and crucial step for accurate CCS determinations [27,29], and we typically use the Agilent ESI Low-concentration Tune Mix. Calibration was performed initially using CCS values provided within the Bruker software. However, a list of slightly different CCS values for the Agilent ESI Low-concentration Tune Mix compounds was recently published by the ion mobility community [29]. Thus, multiple CCS values exist in the literature for the primary tune mix standards, and the community has recognized the need to extract a consensus value [27], but this has not yet happened. Thus, we calculated CCS values for our standards using both primary calibration lists and report these in Tables S1 and S2 (Excel File) until a more complete community vetting of the calibration values can be completed. The average relative standard deviation of triplicate measurements for all measured authentic standards was 0.1026%. 

#### 2.1.2. Comparison of CCS Values with Published Data

We compared our CCS values to a previously published library from Pacific Northwest National Laboratory (PNNL) using DTIMS [29,30]. There were 12 common entries: four fatty acids and nine phenolic acids. The fatty acid measurements had a maximum difference of 0.71%, but interestingly the fatty acids were all uniformly measured here to have a smaller CCS value compared to the PNNL database [30]. The average and relative standard deviation difference was 0.53% (±0.16%). The reported phenolic acid CCS values from PNNL were all larger with the average percent difference of 2.10% (±0.74%). The higher resolution of TIMS relative to DTIMS should result in greater precision in the CCS measurements, while the accuracy of the TIMS determination would depend on accurate calibration.

#### 2.1.3. Isomer Comparisons

Isomer differentiation is a primary motivation for inclusion of IMS in our multi-dimensional metabolomics platform as it is often not achievable by UHPLC-MS alone. Comparisons of multiple groups of isomers were performed after the creation of the CCS library. The largest set of isomers analyzed was the monohydroxyflavones which included seven different isomers. These compounds were used to compare isobaric structural changes and positional variance of the hydroxyl group on CCS values. Triplicate CCS measurements were performed in a broad survey mode using 0.60–1.50 1/K_0_ mobility range and 90 ms ramp time that resulted in an approximate TIMS resolution of 40. Higher TIMS resolutions are easily obtainable, and higher resolution experiments were performed used 0.68–0.76 1/K_0_ mobility range and 400 ms ramp time that yielded an approximate resolution of 120 for the monohydroxyflavones. The CCS apex of the peaks were recorded, and their RSDs were determined to range from 0.09% to 0.26%. 

Resolution is a measure of the separation of two components within a mixture and defined for ion mobility in Equation (2):

Resolution = ΔK_0_/W_K0_ = ΔCCS/W_CCS_, (2)

where ΔK_0_ is the difference in ion mobilities, W_K0_ is the average peak width in mobility units, ΔCCS is the difference in collisional cross sections and W_CCS_ is the average peak width in CCS units.

**Figure 3:**
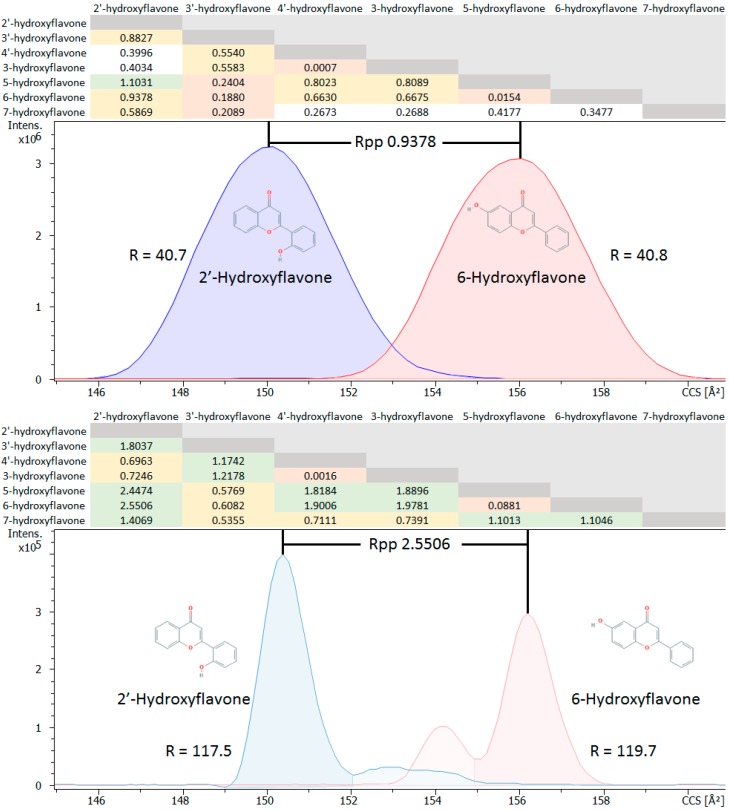
Comparisons of the TIMS resolving power for all combinatorial separations of seven measured isomeric monohydroxyflavones based upon the resolution peak to peak (Rpp) equation from Dodd et al., 2017 [31]. The table shows the calculated R_pp_ for distinguishing CCS values of various isomers monohydroxyflavone pairs using TIMS. Isomer CCS Rpp comparisons are at the intersections of the table’s compound names across the top with the compound names along the left side. Rpp > 1 (green), 1 > Rpp > 0.5 (yellow), 0.5 > Rpp > 0.25 (white), and 0.25 > Rpp > 0 (red). The blue and red traces below the table are extracted ion mobility spectra (EIMs) of 237 m/z and represent the mobilities of 2′-hydroxyflavone and 6-hydroxyflavone respectively. Ten of the 21 possible combinatorial separations of the monohydroxyflavones could be resolved with a Rpp ≥ 0.5 or approximately half height with a TIMS resolution of approximately 40, and 19 of the 21 combinatorial possibilities of the monohydroxyflavones could be resolved with a Rpp ≥ 0.5 and TIMS resolution of ≥ 117.

A resolution of 1 represents baseline separation at 4σ (i.e., peak width 4 times the standard deviation or ± 2σ and confidence level of 95.4%), and a resolution of 0.5 represents resolution at approximately half height or 2σ. Thus, a matrix was established, and resolution values were calculated for all possible combinatorial separations of all monohydroxyflavone pairs (Figure 3) obtained at TIMS resolutions of 40 and 117. The calculated resolution values indicate that 10 of the 21 combinatorial monohydroxyflavone pairs (almost 60%) can be resolved at half height using broad range experimental TIMS parameters (0.60–1.50 1/K_0_) inclusive of a rapid ramp time of 90 ms and a modest resolution of approximately 40. These parameters produce lower resolution but higher sampling rate. However, increasing the ramp time (400 ms) and narrowing the mobility scan range (0.68–0.76 1/K_0_) increases resolution for differentiating isobaric monohydroxyflavones. All but two, i.e., 19 of 21 or 90%, of the combinatorial monohydroxy possibilities could be separated using a TIMS resolution of ≥ 117. These results emphasize the added value of TIMS in differentiating isobaric and isomeric compounds and the results would be expected to be similar or better for other larger polyhydroxy isomeric compound classes.

**Figure 4:**
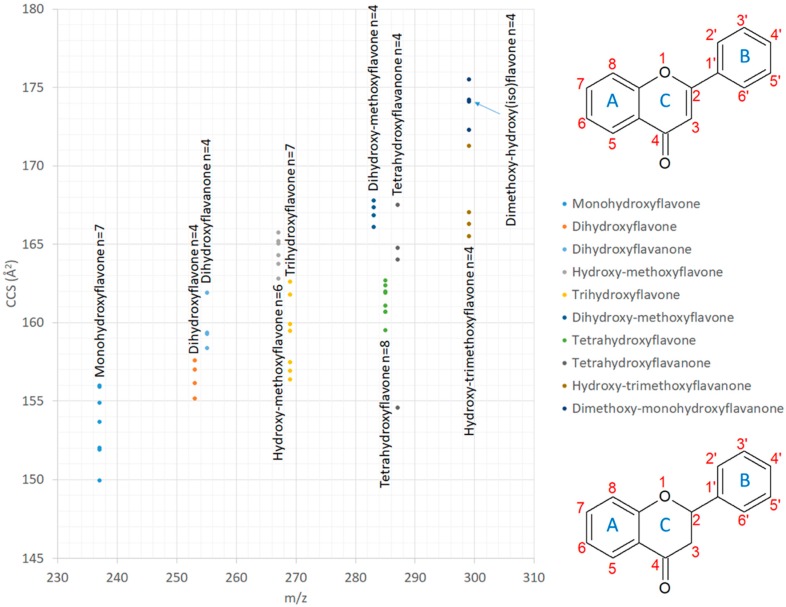
Relative mobilities of several flavonoid isomer classes selected from Figure S1 and general ring structures for flavones (top) and flavanones (bottom) with rings and functional positions labeled. The m/z is indicated on the x-axis and the CCS on the y-axis. The *n* value represents the number of isomers in each group. Many of the isomers have different CCS and could be differentiated. Isomers with the same CCS would need to elute at different times from the UHPLC to be differentiated. General ring structures for flavones and flavanones.

The relationship between CCS and m/z is well established as illustrated in Figure S2 for fatty acids. However, small changes in the spatial structure can often have significant impacts on CCS. For example, the tetrahydroxyflavones differ only by a double bond and two hydrogens in the central ring structure relative to tetrahydroxyflavanones, yet the tetrahydroxyflavanones (average CCS of 162.94 Å^2^) on average have a CCS 2.01 Å^2^ larger than the tetrahydroxyflavones (average CCS of 160.95 Å^2^). See Figure 4. This is likely due to the planar orientation of the flavone C ring attributed to the SP2 hybridization of the C2 atom associated with the double bond. The C ring can then be in-plane with both the A and B rings reducing its overall spatial geometry with the constraining conjugated bonds between C2 and C3 with the attached B ring benzene. In contrast, the orientation of a flavanone, which has an equatorial attachment of the B ring to C2 and no constraining conjugated bonds, allows for free movement and rotation of the B ring causing an increase in average spatial conformation.

### 2.2. Ion Mobility Spectra Interpretations

Extracted ion mobility (EIM) spectra for the expected [M-H]^−^ ions and base peak ion mobility (BPM) spectra containing all adducts and aggregates were smoothed. Mobility peak apices were used to calculate the reported average CCS values. The BPM spectra were often complex and contained several adducts and aggregates making the assignment of the [M-H]^−^ challenging or at times impossible. Thus, CCS values are reported only for annotated peaks of high confidence where the m/z and isotopic pattern matched that of the annotated adduct. Many of these adducts were also found in the mass spectra of a related library [23]. Some annotation challenges could be overcome using EIM spectra or a new TIMS fragmentation method using an increased TIMS Δ6 voltage described in Section 2.3. Relying on the most intense mobility peak was not always found to be a reliable method for ion annotation. 

**Figure 5:**
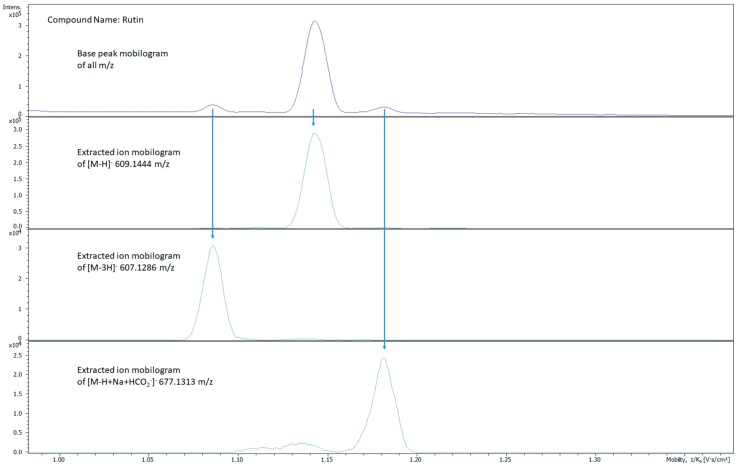
Comparison of the base peak and extracted ion mobility spectra of rutin ions demonstrating the complexity of an ion mobility spectrum that can result from a single compound. TIMS was able to separate three prominent ions present. The top BPM shows all the ions and their relative intensities. The arrows show how the BPM spectrum corresponds with the EIM spectra. The three ions present are: [M-H]^−^ 609 m/z, [M-3H]^−^ 607 m/z, and [M-H+Na+HCO_2_^−^]^−^ 677 m/z.

A series of rutin ion mobility spectra provided in Figure 5 illustrate the explainable complexity of its BPM spectrum. The top BPM spectrum shows more than a single peak for rutin. The assignment of the correct structure to each peak and related CCS is not obvious. The expected [M-H]^−^ with a m/z of 609 has a clear high intensity peak. A sodium formate adduct ion ([M-H+Na+HCO_2_^−^]^−^) at 677 m/z and an oxidized ion [M-3H]^−^ at 607 m/z were also observed. The oxidized ion formation has been previously described [23]. While adducts can be reduced, they cannot be avoided. 

### 2.3. In-TIMS Ion Manipulation

The TIMS tunnel in the timsTOF Pro^TM^ instrument is a two-stage device that includes ion accumulation followed by TIMS analysis. Bruker OtofControl software enables users to optimize the concentration of the ions in the analyzer, sensitivity, duty cycle, and/or resolution dependent on user defined experimental goals and applications. When ions are transferred from the accumulator to the analyzer portion of the TIMS tunnel, the ion velocity can be controlled by adjusting the transfer voltage parameter (TIMS Δ6). Increasing the TIMS Δ6 parameter results in increased ion velocities and increased collisional energies when ions collide with the drift gas. Moderate increases in the TIMS Δ6 can result in ‘in-TIMS’ fragmentation and can often dissociate adduct and aggregate ions to simplify the BPM while even higher energies can begin to fragment the molecular ions. 

**Figure 6:**
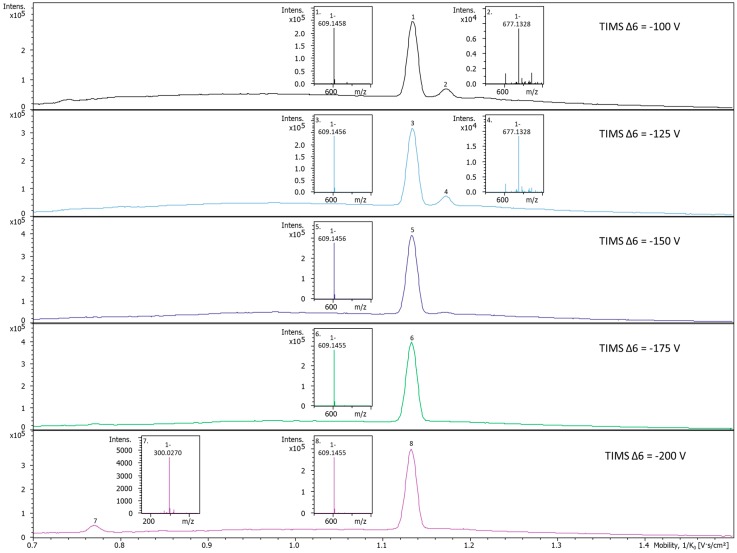
Rutin ion mobility spectra and MS spectra change with increasing transfer voltage parameter (TIMS Δ6) voltages. Increases of the TIMS Δ6 voltage cause changes to the ion mobility spectra of rutin. The 677 m/z adduct is no longer present when the TIMS Δ6 is increased to −175 V, and at −200 V, fragmentation of rutin occurs and a new aglycone ion at 300 m/z appears. This illustrates that increases in the TIMS Δ6 voltage can create collisions within the TIMS tunnel before TIMS analysis. This can be useful for the identification of adducts, reduction in space charge effects, and/or fragmentation of compounds for a three-step fragmentation method using TIMS Δ6, in-source CID, and collision cell CID.

**Figure 7:**
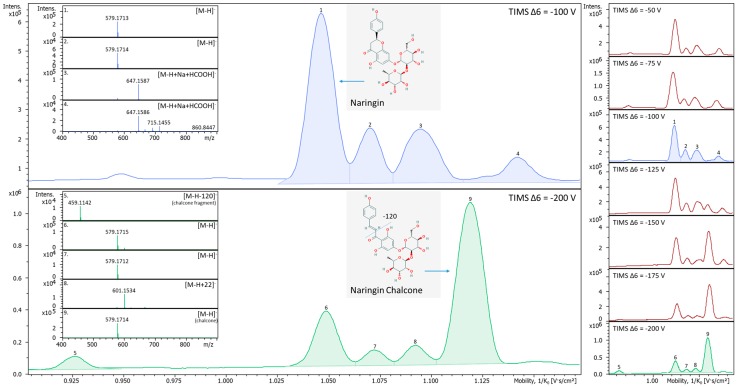
Variations in naringin ion mobility spectra and MS spectra with increasing TIMS Δ6 voltages (right part of the figure). At the default setting of −100 V (blue trace, top left), there are at least four different ion species observed in the ion mobility spectrum indicated as peaks consisting of [M-H]^−^ (peak 1), maybe a slightly larger or elongated conformer of [M-H]^−^ (peak 2), and sodium plus formic acid adduct ([M-H+Na+HCO_2_^−^]^−^, peaks 3 and 4. The trace also contained additional unknown peaks and a dimer that are not shown. A peak emerges at 1.119 1/K_0_ as the TIMS Δ6 voltage is increased from −125 V gradually to −200 V (Green trace, bottom left). Peak 9, shown in the green trace, is an ion that emerged with greater TIMS Δ6 voltage and was not found initially during the analyses. The new peak (peak 9) has been putatively annotated as naringin chalcone. A chain breakage of the naringin likely occurred with the higher energies from the TIMS Δ6 voltage resulting in opening of the C ring and elongated naringin to naringin chalcone. The −120 ion is likely the further fragmentation of the chalcone chain breakage forming a separate mobility peak 5.

Several examples are provided that illustrate the utility of using the TIMS Δ6 parameter in spectral interpretation. These include rutin (Figure 6 and Figure S3), naringin (Figure 7), chrysin (Figure S4), and naringenin (Figure S5). Rutin adducts are reduced with higher voltage transfers, and if too high, the compound fragments. The CCS of naringin increased without any m/z change when the TIMS Δ6 voltage exceeded the default setting of −100 V and going from −125 through −200 V, suggesting the opening of the central ring structure to form naringin-chalcone which increases the size of the analyzed ion in the TIMS drift region. Chrysin did not form as many adducts. Thus, high voltages do not influence the ion mobility spectra until enough energy is applied to fragment the molecule. In summary, the TIMS Δ6 voltage can be adjusted to help identify observed ion mobility spectral peaks.

### 2.4. CCS Matching with Authentic Standards

We analyzed a mixture of authentic standards by direct infusion TIMS-MS and UHPLC-TIMS-MS to evaluate the robustness of the CCS measurements and matching. The mixture included rutin, naringin, naringenin, chrysin, and 6-hydroxyflavone. These compounds include a diverse mixture of hydroxyl, methoxy, and glycosylated flavones and these are commonly used in our lab as a quality control mixture to monitor UHPLC and MS performance. We used a TIMS ion current control (ICC) of 2–5 Mio to prevent potential space charge effects that could alter the measured CCS during the TIMS experiments. The resultant CCS data are included in Table 1 and the compounds separated within the same analysis. The difference in the direct infusion measured CCS values and the library CCS values had an average percent difference of 0.16557% for all authentic compounds. 

Authentic standards were further spiked into a plant root extract and analyzed by UHPLC-TIMS-MS to test the robustness of the CCS measurements and matching within a representative metabolomics sample and to determine if significant matrix affects would alter the CCS measurements. The results were compared to a sample of the same authentic standards without plant matrix. The results in Table 2 reveal an even lower difference then for the direct infusion experiments, and demonstrate the robust accuracy of the measurements. The matrix had a negligible effect. The accuracy was maintained.

### 2.5. CCS Value Matching with Plant Extracts

**Figure 8:**
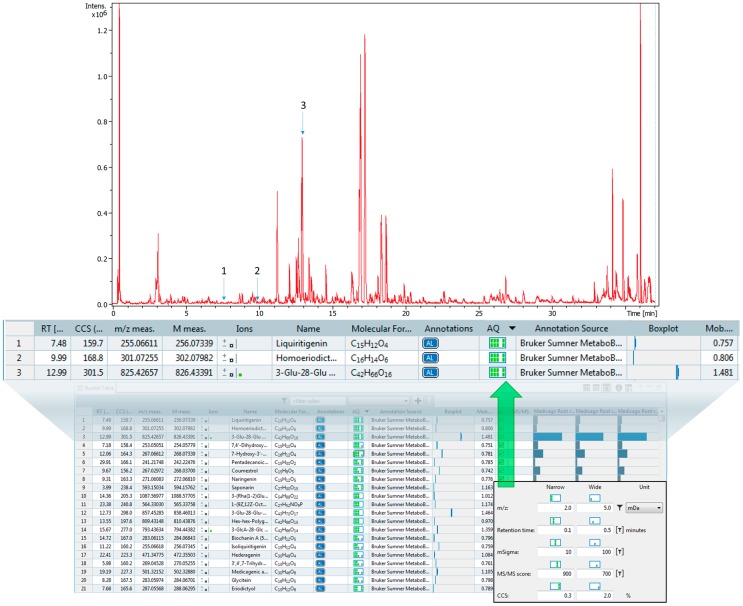
A representative matching report using Bruker MetaboScape software. The annotation quality (AQ) column shows a color coded matching confidence of each of the parameters used in the search (from left to right): accurate mass, retention time, isotopic pattern (mSigma), MS/MS fragmentation, and CCS. Each column has a set of customizable matching parameters. If the first matching criteria is met the square is grey, if the narrower criteria is met both squares are green. The CCS matching is a new orthogonal data feature that expands the confidence in metabolite identifications. The bottom square alone that is grey represents a matching of less than 5% variation between the library and the measured value. Both squares green represents a matching less than 2% variation between the library and the measured value.

To further demonstrate the utility and application of our CCS library we analyzed and processed plant root extracts. *Medicago truncatula* roots were extracted with a mixture of 80% methanol and 20% water and analyzed using UHPLC-TIMS-QTOF-MS. Analyzed extracts of *M. truncatula* roots were processed using MetaboScape. Spectral matching of the root data to library data was performed using exact mass, isotopic patterns, MS/MS, and CCS values. The matching scores are easily visualized with an annotation quality (AQ) graphic reported within the MetaboScape software. A total of 151 compounds in the *M. truncatula* extract were matched with the plant compound library [24] based on accurate mass, and 40 of the 151 were also matched with CCS values. The additional orthogonal CCS data matching provides increased confidence in the identification based upon the Metabolomics Standards Initiative criteria [21]. The data are provided in Table S3 and the processing results are illustrated in Figure 8.

CCS matching in the above experiment was performed using multiple thresholds; i.e., CCS matching score with a moderate confidence matching of ΔCCS ≤ 2.0% and a high confidence matching ≤0.3%. This is within the range of other IMS literature reports of <2% ΔCCS [26]. It is possible that new calibration methods or calibrant compounds could improve the reproducibility even further. These matching scores are based on the apex of the mobility peaks and do not reflect the resolving power of the peaks. We used ion charge control (ICC) to prevent oversaturation of the compounds during the mobility analysis which reduces the accumulation time, and therefore the ion concentration, when ions are detected beyond an intensity threshold.

## 3. Materials and Methods 

### 3.1. Chemicals

Authentic standards were acquired from Sigma-Aldrich (St. Louis, MO, USA), Indofine (Hillsborough, NJ, USA), Chromadex (Los Angeles, CA, USA), PhytoLab (Vestenbergsgreuth, Germany), Supelco, A-APIN, Calbiochem (Danvers, MA, USA), Quality Phytochemicals LLC (East Brunswick, NJ, USA), Sumner Lab, Richard Dixon Lab, and/or Tom Mabry Lab. Burdick & Jackson LCMS grade water and acetonitrile were purchased from Honeywell. ESI-L Low Concentration Tune Mix was purchased from Agilent technologies (Santa Clara, CA, USA). Formic acid 99.5+%, Optima™ LC/MS grade, was purchased from Fisher Chemical (Pittsburgh, PA, USA). The aqueous solvent used for UHPLC contained 0.055–0.1% formic acid. Initially 0.1% formic acid was used, but it was lowered to 0.05% to reduce the presence of adducts. 

### 3.2. UHPLC-ESI-TIMS-QTOF-MS

#### 3.2.1. UHPLC 

Authentic standards were analyzed in triplicate on different days. Authentic standards were suspended in 80% methanol at concentrations ranging from 10–50 ppm and placed in autosampler vials with 300 µL reduced volume inserts. Two microliters of authentic standards were injected onto a Waters UPLC system using a BEH C18 column, 2.1 × 150 mm, 1.7 μm particles that was operated at 60 °C. The flowrate was 0.560 mL/min and gradient elution performed. A linear gradient was performed using binary solvents (solvent A composed of 0.1–0.05% formic acid in water and solvent B composed of 100% acetonitrile). The gradient started at an initial ratio of 95:5% (A:B) and was linearly ramped to 30:70% over 30 min. The gradient was then ramped linearly to 5:95% in 0.1 min (from 36.00 min to 36.10 min) and held at 5:95% for 4 min. Afterwards, the gradient was immediately returned to 95:5 and allowed to re-equilibrate for 4 min. During this re-equilibration time a 20 μL of Agilent ESI-L low concentration tune mix was introduced into the system via a sample loop and 6-way valve for internal calibration. 

#### 3.2.2. ESI-TIMS-QTOF-MS

The Waters UHPLC was connected to a Bruker timsTOF Pro™ instrument equipped with trapped ion mobility coupled to a hybrid quadrupole, time-of-flight mass spectrometer; i.e., UHPLC-ESI-TIMS-QTOF-MS. Ions were generated in the negative electrospray ionization mode. The ESI source used 10 L/min of drying gas at a temp of 250 °C. The drying gas also served as the concurrent TIMS gas. The drying gas was generated by a Parker Balston Model N2-80A membrane based nitrogen generation system operated at an exit pressure of 100 p.s.i. and measured to be composed of 99.4% nitrogen and 0.6% oxygen. The ESI also used a 3500 V capillary voltage, and a 3.0 bar nebulizer pressure. Following the recommended reporting for ion mobility mass spectrometry measurements [27] all user controllable TIMS parameters are reported here. The TIMS cartridge tunnel in pressure was set to 2.50 mbar and tunnel out pressure was 0.74 mbar. The TIMS ion funnels used a fixed radiofrequency of 840 kHz with 275–350 Vpp amplitude depending on the analytes mass. A higher RF amplitude was used for larger compounds and optimized using Agilent tune mix. For maximum sensitivity, compounds were analyzed in different groups based on their m/z value: 200–300 m/z used 275 Vpp, 300–600 m/z used 300 Vpp, and 600+ m/z used 350 Vpp. For routine sample analyses a single RF amplitude is sufficient. Our metabolomics analyses used a funnel 1 RF of 250 Vpp. TIMS inverse reduced mobility (1/K_0_) data were collected over a range of 0.4–1.8 1/K_0_. To prevent TIMS tunnel saturation and space charge effects during TIMS analyses, the ion charge control (ICC) software option was used to adjust the accumulation time of the ions in the first segment of the TIMS, and the ICC set between 2–5 million (Mio). Mass spectral data were collected on the QTOF-MS using a scan range of 100–1500 m/z.

#### 3.2.3. Calibration

Ion mobility and m/z measurements were both externally calibrated using Agilent ESI-L Low Concentration Tune Mix before batch analyses of samples. Batch analyses times were at most 48 h and after this time, the instrument was recalibrated. In addition, a 20 μL aliquot of Tune Mix diluted 1:30 was injected at the end of each analysis and used for internal sample calibration during data processing. The mass and ion mobility were calibrated with calibrants and analyte values interpolated. At least three calibration points were used for a linear TIMS calibration fit. The instrument was externally calibrated to the Bruker default CCS values and the data were internally recalibrated with calibrant injected at the end of each sample analysis. CCS values are reported using both the Bruker default and more recent community calibration values which were slightly different (Tables S1 and S2) [29].

### 3.3. Plant Extract Preparation

Plant extracts were prepared from *Medicago truncatula* Jemalong A17 root. The tissues were harvested in liquid nitrogen, lyophilized, and ground into a fine powder using a mortar and pestle. Dry tissues were stored at −80 °C until extracted. 10 mg of *M. truncatula* dried root tissue was extracted with 1 mL of a mixture of 80% methanol and 20% water. The tissues were vortexed for 2 min, sonicated for 5 min, centrifuged at 4000× *g* for 40 min, and the supernatant collected.

### 3.4. Data Processing and CCS Determination

Data were processed using a pre-release version of Bruker’s MetaboScape 5.0 and DataAnalysis 5.2 software. Mobility and m/z were calibrated using at least 3 calibration points from the Agilent Tune Mix (302, 602, 1034 m/z). UHPLC peaks for the authentic standards were visually selected from their base peak chromatograms (BPC). The expected m/z corresponding to the [M-H]^−^ ion was then used to generate an extracted ion mobility (EIM) spectrum. The ion mobility spectrum was smoothed using a single cycle Savitzky Golay algorithm, 0.005 [V*s/cm^2^] (10 points). Ion mobility spectral peak picking parameters used a sensitivity of 97%, relative intensity threshold of 15%, and minimum peak valley of 10% to generate a table of observed ion mobilities. The software calculated the CCS value from the maximum m/z in the ion mobility spectrum. These peaks were then investigated for the [M-H]^−^ and the peak containing the highest “purity” or a single ion species based on the mass spectra of the peaks mobility range was selected as the true [M-H]^−^ and the CCS value was calculated. CCS data for three analyses per compound were transferred to Microsoft Office Excel where the statistical averages, standard deviations, and relative standard deviations were calculated and recorded for each of 146 compounds detected (Tables S1 and S2).

### 3.5. TIMS Δ6 Adduct Investigations

Adduct formation was investigated using different transfer settings within the TIMS cartridge and more specifically the TIMS Δ6 voltage. The range in the TIMS Δ6 voltage started at relatively low voltage differences (100 V) and was gradually increased (up to 200 V) during direct infusion experiments of single compounds. Segments of the acquisition stepped through increasing voltage differences resulting in increased transfer speeds and collision energies with the drift gas. Data were processed using DataAnalysis visualizing BPM and EIM of the [M-H]^−^ to determine relative intensity changes between different voltage differences of each segments.

## 4. Conclusions

We report here a CCS library focused on plant natural products and specialized metabolites that contains 146 compounds, 343 CCS values, and 29 isomers annotated (various hydroxylated and/or methoxylated and/or glycosylated flavonoids and isoflavonoids). This library is expected to improve metabolite identification confidence and compound identification annotation quality scores through matching of additional orthogonal CCS values included in the library. We also report a method to simplify the spectral complexity of the ion mobility spectra whereby putative adducts and aggregates are dissociated using an increased TIMS Δ6 voltage and MS analyses to verify structural changes. We also found that there were no significant matrix affects observed (less than the standard deviation of the measurements) on the measured CCS as illustrated with a group of standards analyzed by direct infusion and UHPLC-TIMS and spiked within a plant extract. TIMS provides robust, reproducible CCS data with isolated compounds, complex compound mixtures, and compounds within a biological matrix. UHPLC-TIMS-MS/MS provides a four-dimensional metabolomics platform which improves metabolomics’ depth-of-coverage and metabolite identification confidence.

## Figures and Tables

**Table 1:** Differences of CCS values between the compounds analyzed by direct infusion and by UHPLC-trapped ion mobility spectrometry coupled to tandem mass spectrometry (UHPLC-TIMS-TOF-MS). This table uses Stow et al. 2017 [29] Calibration reference.

	UHPLC-TIMS-MS Library CCS ^1^	Direct Infusion Mixture CCS	CCS % Difference
Rutin	231.05	232.37	0.56679%
Naringin	215.92	215.75	0.07876%
Naringenin	163.01	162.99	0.00818%
Chrysin	156.14	156.15	0.00854%
6-hydroxyflavone	155.91	155.61	0.19688%
Average	-	-	0.16557%

**Table 2:** Differences of CCS analyzed with and without plant matrix. This table uses Stow et al. 2017 [29] calibration reference.

Compounds	CCS without Matrix	CCS with Matrix	CCS % Difference
Rutin	232.56	232.64	0.03583%
Naringin	215.66	215.90	0.11431%
Naringenin	162.91	162.95	0.02455%
Chrysin	156.14	156.25	0.07042%
6-hydroxyflavone	156.05	155.94	0.07265%
Average	-	-	0.06355%

## Supplementary Materials

The following are available online at https://www.mdpi.com/2218-1989/10/1/13/s1, Table S1: Cumulative ^TIMS^CCS_N2_ values for 146 authentic plant natural products measured and included the Sumner-Bruker library using the Bruker calibration values for the Agilent Tune Mix, Table S2. Cumulative ^TIMS^CCS_N2_ values for 146 authentic plant natural products measured and included the Sumner-Bruker library using the Stow et al. calibration values [29] for the Agilent Tune Mix, Table S3. UHPLC-TIMS-MS/MS profiling results of *M. truncatula* root extracts in which compound identifications were based upon Rt, exact mass, isotopic patterns MS/MS and ^TIMS^CCS_N2._, Figure S1. Comparison of mass, molecular weight (MW, x-axis) and average CCS (y-axis) of various compounds, Figure S2. Fatty acids m/z compared to average CCS, Figure S3. Rutin ion mobility spectra and MS spectral changes associated with increasing collision energies, Figure S4. Chrysin and naringenin do not have altered ion mobility spectra or fragmentation with the increased TIMS Δ6 voltages.
