# Supplementary material for: Generation of a Collision Cross Section Library for Multi-Dimensional Plant Metabolomics Using UHPLC-Trapped Ion Mobility-MS/MS
Source: Metabolites. 2019 Dec 24;10(1):13. doi: 10.3390/metabo10010013 (PMC7023306; doi:10.3390/metabo10010013)
Supplement: Supplementary file 1 [file metabolites-10-00013-s001.zip › supplementary-figures.pdf]

Supplemental Figures:

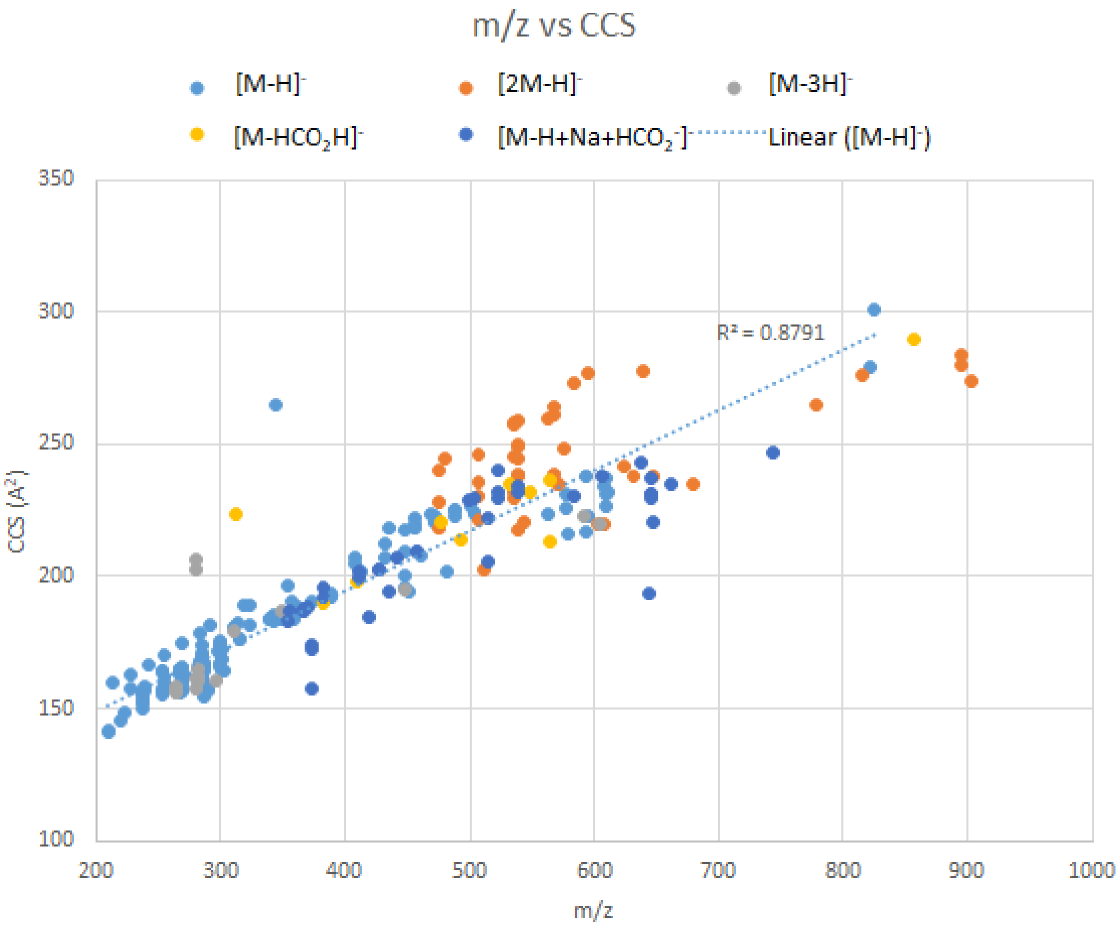

Figure S1. Comparison of mass-to-charge (m/z, x-axis) and average CCS (y-axis) of the compounds measured. The overall $R^2 = 0.8782$ illustrates a clear correlated positive trend of m/z and CCS.

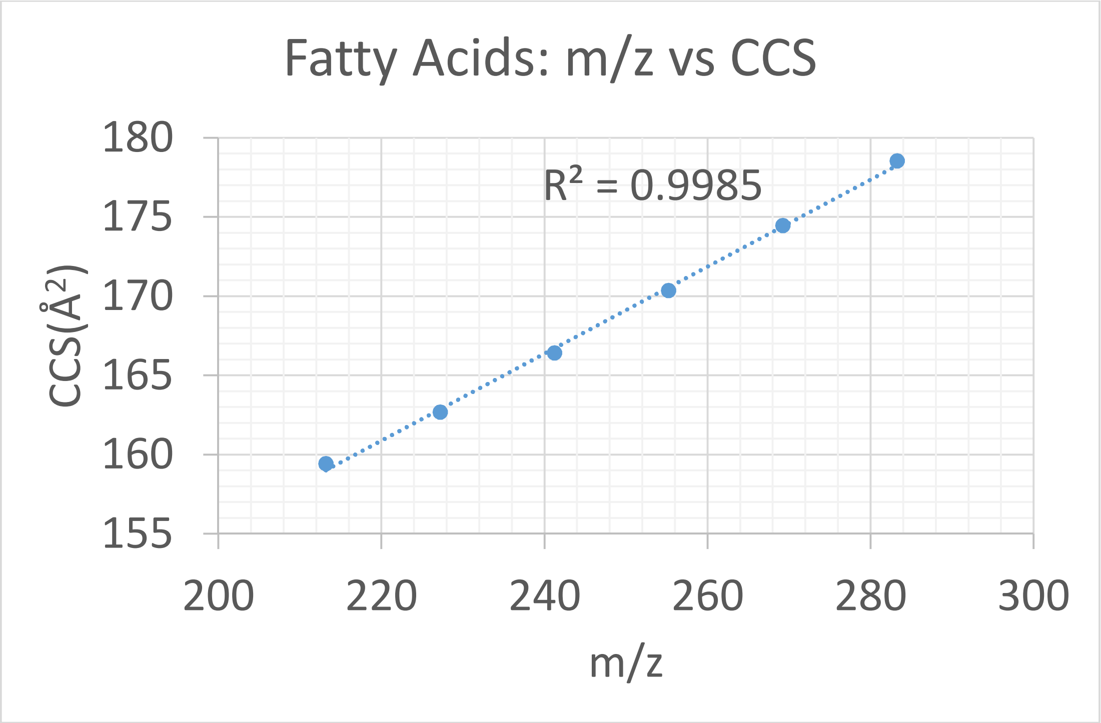

Figure S2. Fatty acid m/z compared to average CCS values. The fatty acids have a higher R^2 linear fit than the other compounds tested, but the number of fatty acids tested was modest.

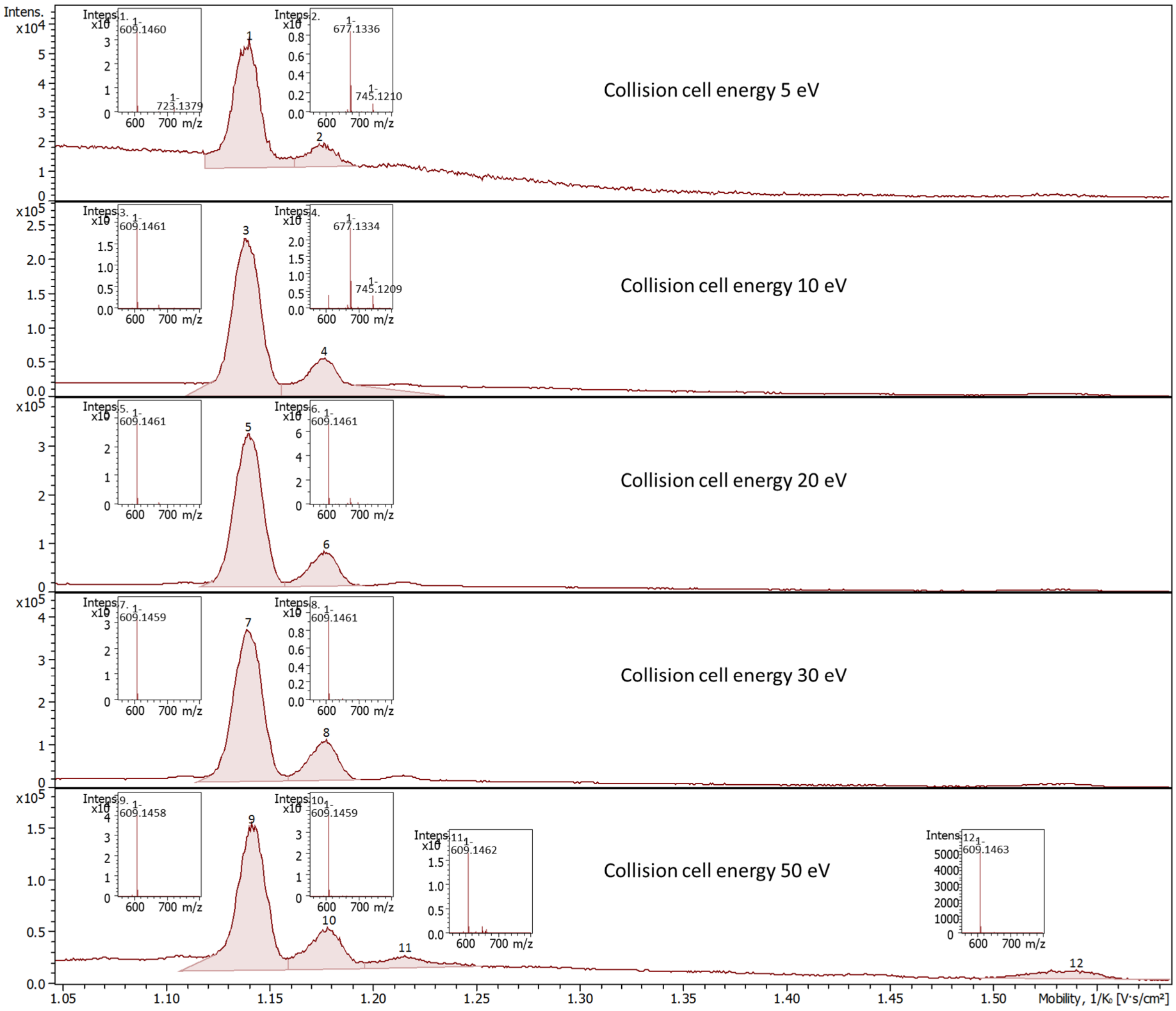

Figure S3. Rutin mobilograms MS spectra changes with increasing collision energies. The collision cell is positioned after the TIMS mobility separation. By increasing the collision energy we can determine what compounds are present within each mobility peak and be confident the additional peaks are adducts of the analyte and not a contaminant. As the collision cell energy is increased the [M-H]$^-$ (609 m/z) ion is found in more of the mobility peaks. This shows the additional mobility peaks are from adducts and adducts can be broken up using sufficient collision energy.

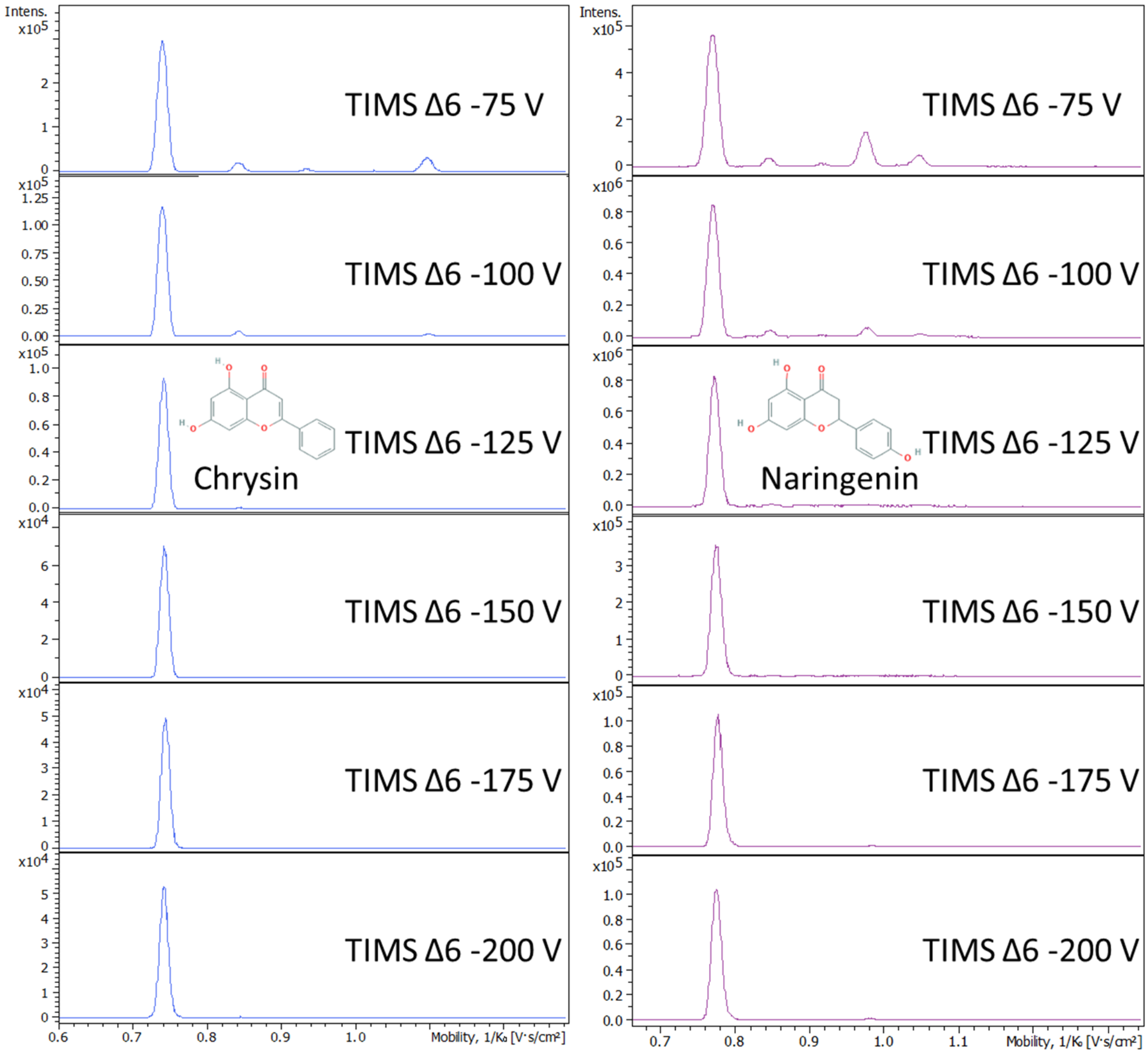

Figure S4. Chrysin and naringenin do not have altered ion mobility spectra or fragmentation with the increased TIMS Δ6 voltages. Increasing the TIMS Δ6 voltage caused adducts to break apart and simplify the mobilogram from the -75 V to -200 V. There is a slight decrease in intensity (y-axis) likely from fragmentation, but no fragment mobility peaks were found.
